# Modulation of Hippo signaling by Mnat9 N-acetyltransferase for normal growth and tumorigenesis in *Drosophila*

**DOI:** 10.1038/s41419-022-04532-2

**Published:** 2022-02-02

**Authors:** Jung-Wan Mok, Kwang-Wook Choi

**Affiliations:** Department of Biological Sciences, Korea Advanced Institute of Science and Technology, Daejeon, 34141 Korea

**Keywords:** Mechanisms of disease, Cell proliferation, Disease model, Drosophila

## Abstract

Hippo signaling is a conserved mechanism for controlling organ growth. Increasing evidence suggests that Hippo signaling is modulated by various cellular factors for normal development and tumorigenesis. Hence, identification of these factors is pivotal for understanding the mechanism for the regulation of Hippo signaling. *Drosophila* Mnat9 is a putative N-acetyltransferase that is required for cell survival by affecting JNK signaling. Here we show that Mnat9 is involved in the negative regulation of Hippo signaling. RNAi knockdown of Mnat9 in the eye disc suppresses the rough eye phenotype of overexpressing Crumbs (Crb), an upstream factor of the Hippo pathway. Conversely, *Mnat9 RNAi* enhances the eye phenotype caused by overexpressing Expanded (Ex) or Warts (Wts) that acts downstream to Crb. Similar genetic interactions between *Mnat9* and Hippo pathway genes are found in the wing. The reduced wing phenotype of *Mnat9 RNAi* is suppressed by overexpression of Yorkie (Yki), while it is suppressed by knockdown of Hippo upstream factors like Ex, Merlin, or Kibra. Mnat9 co-immunoprecipitates with Mer, implying their function in a protein complex. Furthermore, Mnat9 overexpression together with Hpo knockdown causes tumorous overgrowth in the abdomen. Our data suggest that Mnat9 is required for organ growth and can induce tumorous growth by negatively regulating the Hippo signaling pathway.

## Introduction

Proper regulation of cell proliferation and cell death is important for tissue homeostasis and organ growth in developing animals [1]. The growth of tissues and organs is regulated by multiple evolutionarily conserved signaling pathways. The Hippo signaling pathway is a key mechanism for regulating organ size [2–4]. The core of this pathway is a kinase cascade consisting of serine/threonine kinases, Tao-1, Hpo, and Wts [5]. This cascade is activated by a network of various upstream factors, including Crb, Ex, and Mer [6–8]. Activated Wts phosphorylates Yki to block its function as a transcriptional coactivator for the target genes that promote cell proliferation and cell survival. Hence, loss of Hippo signaling or activated Yki can induce aberrant overgrowth of organs to cause cancers [9].

Although Hippo signaling is abnormally regulated in many types of cancers, somatic or germline mutations in Hippo pathway genes have been rarely found in human cancers [10]. Hence, it has been suggested that frequent abnormal Hippo signaling in cancer tissues occurs through molecular defects other than the mutation in Hippo pathway genes. Accumulating evidence indicates that Hippo signaling is regulated by a number of cellular factors. For example, Hippo signaling is affected by various cell junctions, cytoskeletal factors, and cell-polarity complexes [11, 12]. Several signaling pathways, including Wnt and Notch pathways, also affect Hippo signaling [13–15]. Interestingly, Jun N-terminal kinase (JNK) signaling can be anti-tumorigenic or tumorigenic, depending on cellular context. During compensatory proliferation, JNK signaling can promote growth by upregulating Yki [16, 17]. Conversely, JNK signaling can downregulate Yki during cell competition and tissue-growth control [18–20]. These studies suggest that Hippo signaling is regulated in a network of multiple signaling pathways for normal tissue growth. Thus, identification of regulatory factors affecting Hippo signaling is important for understanding the molecular network of the Hippo pathway in normal organ development and tumorigenesis.

Recently, we have found a novel *Drosophila* microtubule-associated N-acetyltransferase (Nat) named Mnat9 [21]. The Nat proteins are enzymes involved in the post-translational acetylation of newly synthesized proteins, but their functions in vivo are largely unknown. Mnat9 plays an essential role in cell survival during organ growth by negatively regulating JNK signaling [21]. Based on the reported roles of JNK signaling in modulating the Hippo pathway, it is an intriguing question whether Mnat9 might interact with the Hippo signaling pathway to regulate organ growth.

In this study, we show that Mnat9 negatively affects Hippo signaling and directly interacts with Mer. Furthermore, we demonstrate that Mnat9 overexpression and reduced Hippo signaling synergistically interact to induce overgrowth. Our data suggest that Mnat9 is required to inhibit Hippo signaling for normal development and that its upregulation can promote tumorous growth.

## Materials and methods

### Fly stocks and culture

Flies were cultured at room temperature (RT) for stock maintenance. All genetic experiments were carried out at 29 °C on standard media using the Bloomington Drosophila Stock Center (BDSC) recipe, unless specified otherwise. All the progenies were counted or analyzed without randomization or blinding. We utilized the Gal4/UAS system for misexpression or downregulation of genes in flies [22]. The following stocks were obtained from BDSC: *GMR-Gal4 (#1104), en-Gal4* (#30564), *UAS-Dcr-2; en-Gal4*, *UAS-GFP* (#25752), *pnr-Gal4* (#3039), *UAS-Dcr-2; pnr-Gal4* (#25758), *UAS-Dcr-2; C96-Gal4 (#25757), UAS-Mer RNAi (#28007), UAS-hpo RNAi (#27661), UAS-kibra RNAi (#28083), UAS-ex RNAi (#28703), UAS-yki-GFP (#28815)*, and *ex-lacZ (#44248)*. *UAS-dicer (Dcr)-2* was used to increase RNAi effects [23, 24]. The following stocks were gained from the Vienna Drosophila Resource Center (VDRC): *UAS-hpo RNAi* (*v7823*, *v104169*), *UAS-mats RNAi (v108080)*, and *UAS-Mnat9 RNAi (v31519, v104497, v49580)*. Other lines were *UAS-Mnat9-HA* (FlyORF), *Kibra:GFP* (a gift from Richard Fehon), *UAS-ex* [25]*, UAS-wts* (a gift from Georg Halder), and *UAS-Crb*^*intra*^ [26].

### Immunoprecipitation

A Midi-prep kit (Macherey-Nagel) was used to prepare high-purity (A260/280 ~1.8) plasmid DNA. For transfection, *Drosophila* S2 cells were mixed with 1–2 μg of plasmid DNA and Effectene (Qiagen) following the manufacturer’s instructions. After 48 h, cells were harvested and lysed using a glass homogenizer with HEPES buffer (0.5% CHAPS) at 4 °C. To prevent protein degradation or dephosphorylation, protease-inhibitor cocktail (Roche) and PHOSTOP (Roche) were used. Samples were centrifuged at 13,000 *g* at 4 °C for 15 min, and the supernatant was used for further experiments. Beads (Surebead, Bio-rad) were precleared for 30 min at RT. Precleared beads were incubated with control rabbit IgG (Genscript-A01008) or Mnat9 antibody (1:100) [21] for 1 h at 4 °C. Antibody-bound beads were incubated with samples for 2 h at 4 °C and washed at least four times before elution.

### Immunohistochemistry

For immunostaining of imaginal discs, third-instar larvae were collected and washed with PBS solution. Dissected imaginal discs were fixed with 4% paraformaldehyde fixative (PFA) for 30 min at RT. After a brief PBS wash, discs were incubated with 0.5% PBT (PBS with 0.5% Triton X-100) for 30 min for permeabilization. Discs were further incubated with blocking solution (10% normal goat serum, 0.3% Triton X-100 in PBS) for 1 h at RT. After blocking, samples were incubated with primary antibodies overnight at 4 °C. Primary antibodies were α-lacZ (1:50, Mouse, DSHB 40-1a), α-GFP (1:500, Chicken, Abcam-ab13970), α-Mer (1:7500, Guinea Pig, a gift from Richard Fehon), α-Ex (1:5000, Guinea Pig, a gift from Richard Fehon), and α-Mnat9 (1:200, Rabbit [21]). Discs were then washed at least three times with 0.3% PBT before treating with secondary antibodies. Secondary antibody staining was done at RT for 1 h. Secondary antibodies were α-Mouse Cy3 (1:600, Goat, Jackson 115-165-003), α-Rabbit Cy5 (1:200, Goat, Jackson 111-175-144), and α-Guinea Pig Cy5 (1:200, Donkey, Jackson 706-175-148). After three times of washing (0.3% PBT), samples were mounted (Vector, H-1000) on slide glasses. For image acquisition, Zeiss LSM 710 confocal microscope was used.

### Cell culture and maintenance

*Drosophila* S2 cell line (DGRC, stock #6) was maintained using M3 + BPYE media (Sigma) supplemented with 10% FBS (Hyclone). Cells were cultured at 25 °C and transferred every 3–5 days. Fresh cells with high viability (>95%) were stored in a liquid-nitrogen container until used for experiments.

### Wing-size quantification

To quantify the size of the anterior and posterior wing compartments, we used the approximate anterior–posterior boundary between the L3 and L4 veins, as shown in Fig. S1A. Wing size was calculated using the ImageJ program.

### Western blotting

For western blotting, protein samples were boiled in SDS sample buffer at 90 °C for 10 min and centrifuged at 12,000 *g*. Samples in supernatants were fractionated by 10% or 12% SDS-PAGE electrophoresis. PVDF membrane was used for protein transfer. Transferred membranes were blocked using 5% BSA in TBST (0.1% tween-20) for 30 min at RT. After blocking, membranes were incubated with primary antibodies (rabbit anti-Mer, 1:1000; mouse anti-MBP, 1:3000, NEB-E8032L) overnight at 4 °C and washed using 0.1% TBST at least three times at RT. After washing, membranes were incubated with secondary antibodies (mouse anti-HRP, Invitrogen #31430, 1:10,000; rabbit anti-HRP, 1:10,000, Invitrogen #31460) for 1 h at RT. Before incubating with Pico-plus ECL (Thermo), membranes were washed using 0.1% TBST at least three times at RT. X-ray film (AGFA) was used for ECL detection.

### Scanning electron microscopy

Quattro S environmental SEM (ESEM, Thermo) was used for image acquisition. One-day-old adult flies were collected and stored at −20 °C for 24 h prior to use for imaging. No pretreatment was done except freezing. Imaging was performed in Kaist Analysis Center for Research Advancement (KARA). Imaging conditions were as follows: temperature: 2 °C, pressure: 730 Pa, humidity: 100%, and HV: 10 kV.

## Results

### *Mnat9 RNAi* enhances gain-of-function effects of Hippo components in the eye

**Fig. 1:**
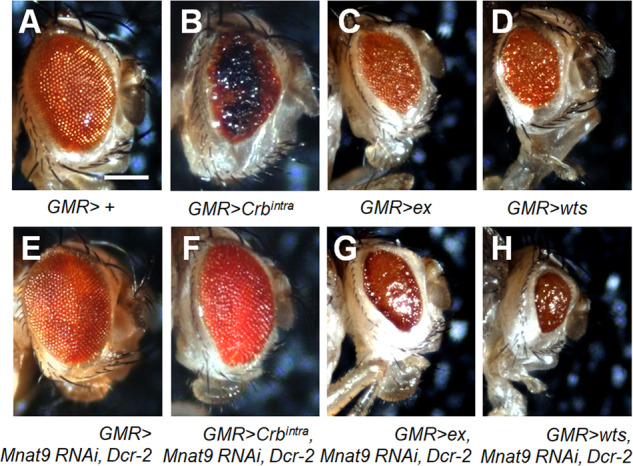
Mnat9 genetically interacts with Hippo pathway components in the *Drosophila* eye. **A** Control normal eye with *GMR-Gal4*. **B**–**D** Eye phenotypes caused by overexpression of the following factors using *GMR-Gal4*: Crb^intra^ (**B**), Ex (**C**), and Wts (**D**), respectively. **E** *Mnat9 RNAi* shows little defect. **F**–**H** Genetic interaction between *Mnat9 RNAi* and overexpressed Crb^intra^ (**F**), Ex (**G**), or Wts (**H**). Crb^intra^ phenotype is suppressed by *Mnat9 RNAi*. However, phenotypes of Ex or Wts overexpression are enhanced by *Mnat9 RNAi*. Scale bar: 200 μm.

To test whether Mnat9 is involved in organ growth by affecting Hippo signaling, we examined genetic interaction between *Mnat9* and *crb* using the Gal4-UAS method [27]. Crb is a transmembrane protein that acts upstream in the Hippo pathway [28]. Overexpression of the intracellular domain of Crb (Crb^intra^) in the differentiating eye disc using *GMR-Gal4* (labeled *GMR* > *Crb*^*intra*^) causes severe roughening in the adult eye (Fig. 1B). Although knockdown of *Mnat9* showed no obvious eye defects in the wild-type background (Fig. 1A, E), it strongly suppressed the eye phenotype of Crb^intra^ overexpression (Fig. 1F). Three different *Mnat9*-*RNAi* lines (*v104497*, *v49580*, and *v31519)* showed similar suppression of the Crb^intra^ eye phenotype. Next, we tested whether *Mnat9* shows genetic interaction with *ex* that acts downstream to *crb*. Ex is known to be downregulated by Crb^intra^ overexpression [29]. As expected, the rough-eye phenotype of Ex overexpression was enhanced by *Mnat9 RNAi* (Fig. 1C, G). Overexpression of Wts, a core kinase of the Hippo pathway, shows similar eye phenotypes as Ex overexpression (Fig. 1C, D). The effects of Wts overexpression were also strongly enhanced by *Mnat9 RNAi* (Fig. 1H).

We also tested this interaction in the wing, using *en-Gal4* that drives Gal4 expression in the posterior-wing compartment (Fig. S1). Wts overexpression by *en-Gal4* (*en* > *wts*) led to a reduction in the posterior region at 18 °C (Fig. S1C). Under the same condition, *Mnat9 RNAi* showed little reduction in the wing (Fig. S1B). However, *Mnat9 RNAi* significantly enhanced the Wts-overexpression phenotype, resulting in a loss of most regions between the longitudinal veins 4 and 5 (Fig. S1D, E, red arrow). These results suggest that loss of Mnat9 leads to activation of Hippo signaling.

### *Mnat9 RNAi* suppresses loss-of-function effects of Hippo components

**Fig. 2:**
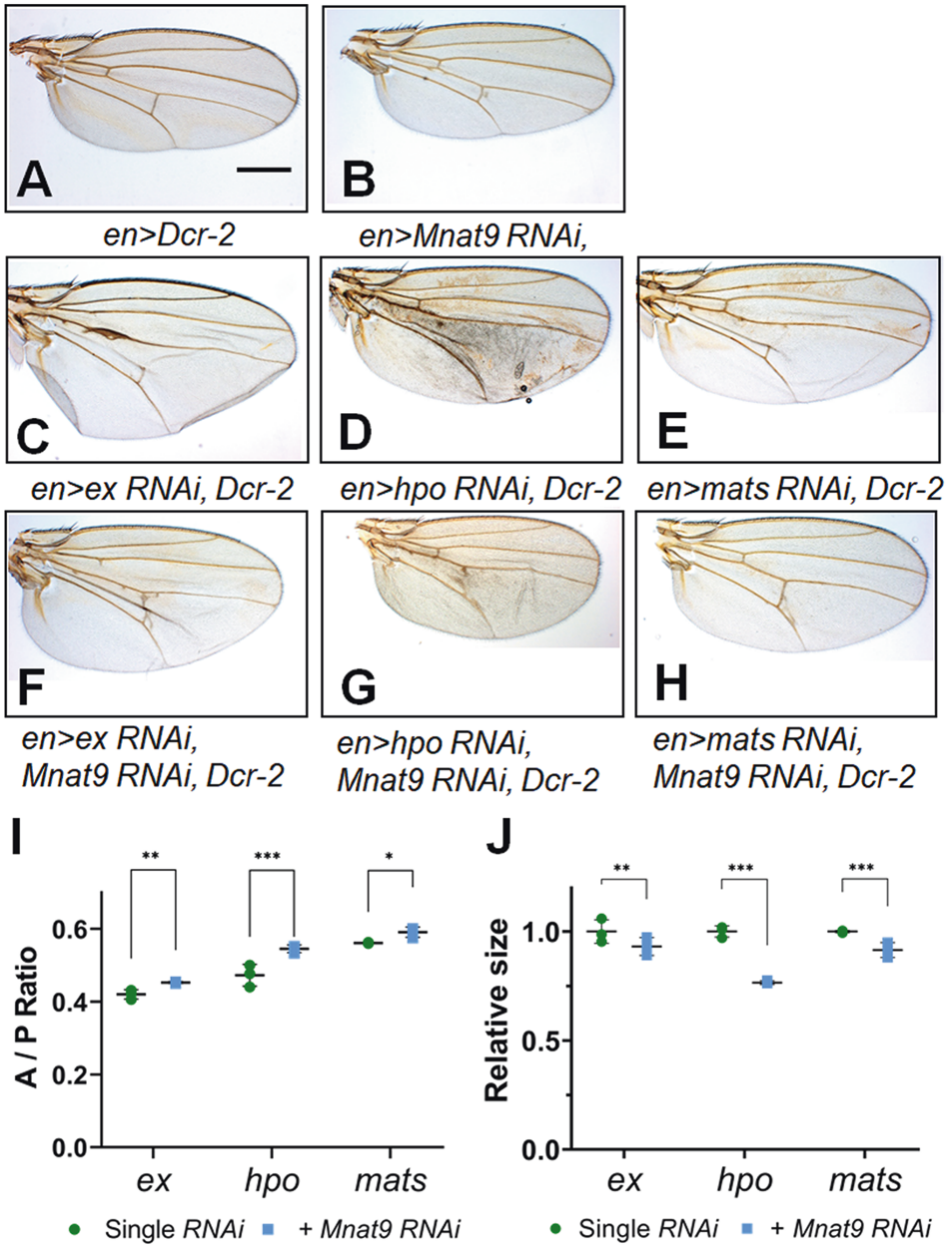
*Mnat9* shows genetic interaction with Hpo components in the wing. **A** *en* > *Dcr-2* control. **B** Knockdown of Mnat9 in the posterior wing domain by *en-Gal4* does not affect the wing size. **C**–**E** Knockdown of *ex* (**C**), *hpo* (**D**), *or mats* (**E**) causes enlarged posterior wing tissue. **F**–**H** Knockdown of *Mnat9* suppresses the phenotypes of RNAi for Ex (**F**), Hpo (**G**), or Mats (**H**). Quantification of the anterior/posterior (A/P) ratio (**I**) and the relative wing size (**J**) from data shown in **C**–**H**. Scale bar: 0.5 mm. *n* = 3 for each genotype and “multiple *t*-test” was used for statistical analysis. *p < 0.05, **p < 0.03, ***p < 0.01. Error bars are standard deviations.

Because *Mnat9 RNAi* enhances the effects of overexpressing Hippo components, we tested whether Mnat9 knockdown can suppress the phenotypes of reduced function of the Hippo pathway. As *ex RNAi* phenotype was more pronounced in the wing than the eye, we chose to examine wings for this genetic test. However, double knockdown of Mnat9 and Ex resulted in lethality at RT and higher temperatures. Because Gal4 activity depends on temperature [30], we performed these genetic interaction tests at 18 °C to avoid the lethal effects of double knockdown. *Mnat9 RNAi* itself did not affect the wing size at 18 °C, although it caused mild extra vein formation near the posterior cross-vein (Fig. 2B). Knockdown of Hippo pathway genes such as *ex, hpo,* and, *mats* with *en-Gal4* led to an enlargement in the targeted posterior region of the wing and partial loss of the posterior cross-vein (Fig. 2C–E). We then examined the effects of the double knockdown of *Mnat9* and one of these Hippo pathway genes. The increased wing-size phenotypes of *ex*, *hpo*, or *mats* *RNAi* were partially suppressed by *Mnat9 RNAi* (Fig. 2F–J). These results suggest that Mnat9 is related antagonistically to Hippo pathway genes in wing growth.

### Wing reduction by *Mnat9 RNAi* is suppressed by reducing Hippo signaling

**Fig. 3:**
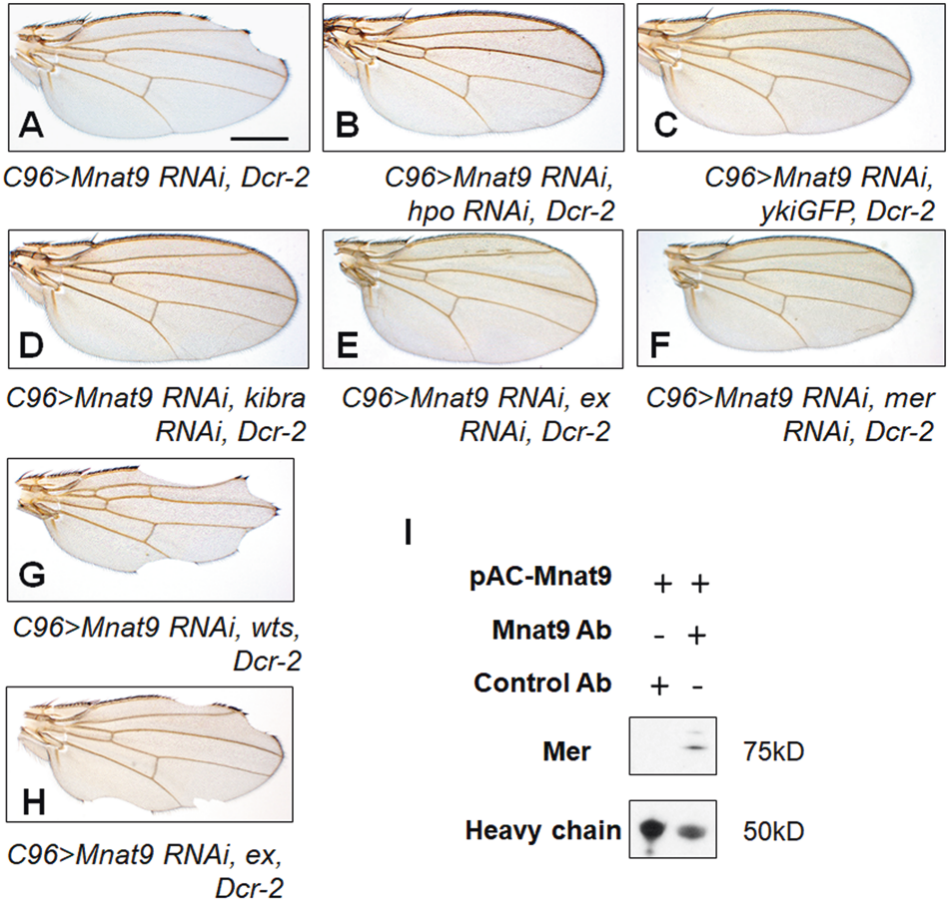
*Mnat9 RNAi* phenotypes are suppressed by knockdown of Hippo pathway components. **A** *C96* > *Mnat9 RNAi* causes wing notching. **B**–**H** Genetic interaction between *Mnat9 RNAi* and Hippo components. *hpo RNAi* (**B**) or *yki* overexpression (**C**) rescues *Mnat9* *RNAi* phenotypes. Knockdown of *kibra* (**D**), *ex* (**E**), and *mer* (**F**) rescues *Mnat9 RNAi* phenotype. Overexpression of *ex* (**G**) or *wts* (**H**) enhances the *Mnat9 RNAi* phenotype. Scale bar: 0.5 mm. **I** Mer is co-immunoprecipitated with anti-Mnat9 antibody but not by control rabbit IgG.

The data above suggest that Hippo signaling may depend on Mnat9 or vice versa. To further characterize their relationships, we tested whether the organ size defect caused by *Mnat9 RNAi* can be suppressed by reducing the Hippo signaling components. *C96-Gal4* drives Gal4 expression in the dorsoventral boundary region of the wing disc that develops to the margin of the adult wing. Knockdown of *Mnat9* using *C96-Gal4* resulted in notching along the wing margin, indicating that Mnat9 is required for wing development (Fig. 3A). In control tests, downregulation of Hippo signaling components such as Hpo and Wts using *C96-Gal4* did not cause notching along the wing margin. Overexpression of *wts* or *ex* resulted in smaller wings with some notching phenotype, while Yki overexpression showed little effect on wing growth under this test condition (Fig. S2).

Next, we examined the combined effects of *Mnat9 RNAi* and altered Hpo components. The notched wing phenotype of *Mnat9 RNAi* was restored by knockdown of *hpo* or overexpression of its downstream factor *yki* (Fig. 3B,C). Furthermore, *Mnat9 RNAi* phenotype was suppressed by downregulation of Hpo upstream factors like *kibra*, *ex*, or *mer* (Fig. 3D–F). Conversely, *C96* > *Mnat9 RNAi, Dcr-2* phenotype was strongly enhanced by overexpression of Wts (Fig. 3G) or Ex (Fig. 3H). Suppression of the *Mnat9 RNAi* phenotype by knockdown of Ex, Mer, or Kibra suggests that Mnat9 may act upstream of Hpo by antagonizing the function of Ex, Mer, and Kibra.

**Fig. 4:**
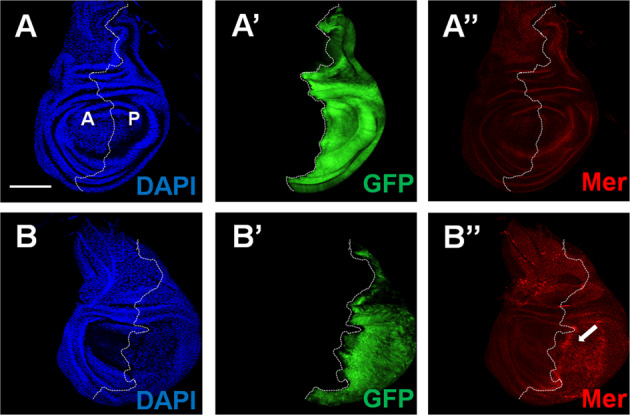
Downregulation of *Mnat9* increases Mer protein level. Wing discs were stained for DAPI, GFP, and Mer, as indicated. **A**–**A**″ Control wing with *en* > *GFP*. **B**–**B**″ Wing discs with *en* > *Mnat9 RNAi, GFP*, and *Dcr-2*. The posterior *en* domain is shown by GFP staining (**B**′). *Mnat9* downregulation by *en-Gal4* increases Mer protein level in the posterior part of the wing disc (**B**″ arrow). Scale bar: 100 μm. Dashed line: anterior (A)–posterior boundary (P).

Recently, we have shown that Mnat9 is associated with microtubules [21]. Mer is also known to bind microtubules [5, 31–34]. Based on the genetic interaction between Mnat9 and Mer, we wondered whether Mnat9 might directly interact with Mer. Immunoprecipitation experiments with S2 cells revealed that Mnat9 co-immunoprecipitates with endogenous Mer (Fig. 3I), suggesting their presence in a protein complex. Because our genetic data suggest that Mnat9 may act upstream of Mer, Ex, and Kibra, we tested if Mnat9 may interact with Mer to downregulate these upstream factors of the Hippo pathway. Indeed, we found that Mer protein level was increased in the Mnat9-depleted posterior part of the wing disc (Fig. 4B″). However, Ex and Kibra-GFP levels were not altered (Fig. S3), suggesting that Mnat9 may have specific effects on Mer without altering the whole Mer/Ex/Kibra complex.

### Overexpression of Mnat9 causes tumorous growth in Hpo-depleted tissues

To determine whether Mnat9 is sufficient to inhibit the Hippo pathway, we tested whether Mnat9 overexpression can induce ectopic Yki activity, using *ex-lacZ* as a reporter for the Yki target gene *ex*. When Mnat9 was overexpressed by using *en-Gal4*, the level of *ex-lacZ* reporter expression or the size of wing disc was not affected in the posterior region of the wing disc (Fig. S3B–B″), indicating that overexpression of Mnat9 alone cannot activate Yki function in the wing disc.

**Fig. 5:**
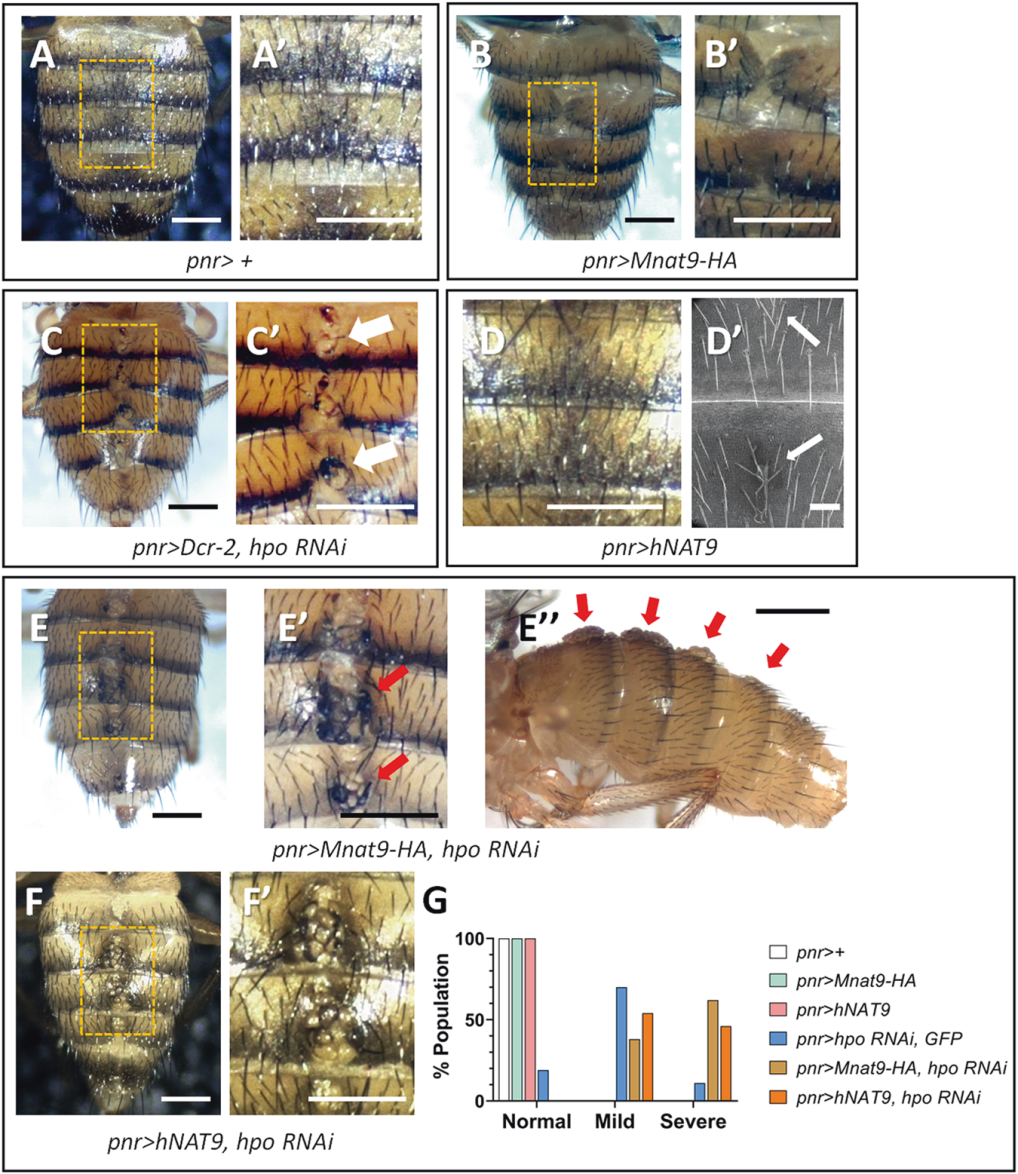
Overexpression of Mnat9 causes tumorous growth in Hpo-depleted tissues. **A**, **A**′ *pnr-Gal4* control. **B**, **B**′ Overexpression of *Mnat9* causes mild cleft along the midline of the abdomen. **C**, **C**′ Knockdown of *hpo* induces mild overgrowth in the medial part of the adult abdomen (white arrows in **C**′). **D**, **D**′ Overexpression of hNAT9 causes no visible phenotype under dissection scope, but SEM images occasionally (40%, *n* = 10) show misoriented bristles at the midline. **E**–**E**″ *Mnat9* overexpression combined with *hpo* knockdown induces tumorous tissues protruding from the midline of the abdomen (red arrows). **F**, **F**′ Similar tumorous growth was also found by hNAT9 overexpression with *hpo RNAi*. **A**′, **B**′, **C**′, **E**′, and **F**′ are enlarged views of the boxed area in **A**–**F**, respectively. **G** Quantification of tumorous phenotypes. Percent (%) fly populations showing normal, mild, or severe abdominal tumorous phenotype (*n* > 50 for each genotype). Mild: 1–3 large cell mass/abdominal segment as indicated by arrows in (**C**′). Severe: >3 large cell mass as indicated by an arrow in (**E**′, **E**″). Scale bar for SEM image (**D**′): 50 μm. Scale bars for other images: 200 μm.

Hence, we tried several Gal4 drivers to identify the regions where Mnat9 overexpression can promote tissue overgrowth. Interestingly, when Mnat9 was overexpressed by *pannier (pnr)-Gal4*, we detected incomplete closing of left and right abdominal hemispheres in about 21% of flies (*n* = 84) (Fig. 5B). *pnr-Gal4* drives gene expression in the dorsal midline of the thorax and abdomen [35], suggesting that Mnat9 overexpression interferes with the dorsal closure of the abdominal hemispheres. However, no tumorous growth was observed in the abdominal midline region by Mnat9 overexpression. Intriguingly, we found that *hpo RNAi* by *pnr-Gal4* causes similar defects in the dorsal closure of the abdomen (Fig. 5C). Further, *hpo RNAi* also resulted in extrusion of tumor-like cell clusters (Fig. 5C–C’). We divided tumorous phenotypes into mild (3 or fewer cell masses/segment) and severe (more than three cell masses/segment) cases. About 68% and 11% of *hpo RNAi* flies showed mild and severe overgrowth phenotypes, respectively (*n* = 54) (Fig. 5G).

Next, we checked whether Mnat9 overexpression in the *hpo RNAi* condition can cause synergistic defects in the abdominal midline. Remarkably, when Mnat9 overexpression and *hpo* knockdown were combined, the abdominal midline showed more severe closure defects and tumorous overgrowth within the cleft (Fig. 5E, E’, G), resulting in the severe tumorous phenotype in 61% of flies (Fig. 5E″, G, *n* = 65, red arrows). These results show that Mnat9 overexpression can induce tumorous growth by a synergistic interaction with the condition of reduced Hippo signaling.

Previously, we have shown that *Drosophila* Mnat9 and the human homolog hNAT9 are functionally conserved [21]. Flies overexpressing hMnat9 by *pnr-Gal4* appeared normal under a dissection microscope. However, images from scanning electron microscopy (SEM) showed some irregular orientation of bristles in the abdominal midline (Fig. 5D, D′, G), suggesting that the midline is not perfectly normal. Next, we tested whether hNAT9 can induce overgrowth in Hpo-depleted tissues. While overexpression of hNAT9 alone caused no overgrowth phenotypes, it resulted in overgrowth along the abdominal midline under the *hpo RNAi* background (Fig. 5F, F′). Hence, Mnat9 and hNAT9 seem to be conserved in their functions to induce overgrowth by interacting with the Hippo pathway.

Mnat9 is a putative N-terminal acetyltransferase based on its conserved acetyl-CoA-binding motif and its enzyme activity in vitro. However, genetic evidence suggested that the enzyme activity of Mnat9 is not essential for its biological function to regulate cell survival [21]. Hence, we tested whether N-terminal acetyltransferase activity is required for the Mnat9 function to interact with Hpo. With *pnr-Gal4*, overexpression of an Mnat9-[AcDel] mutant form deleted in the acetyl-CoA-binding motif did not show any noticeable defects. However, overexpression of Mnat9-[AcDel] along with *hpo RNAi* caused large clusters of overgrown cells (Fig. S5B), as seen with wild-type Mnat9. These results indicate that N-terminal acetyltransferase activity might be dispensable for the interaction of Mnat9 with the Hippo pathway.

**Fig. 6:**
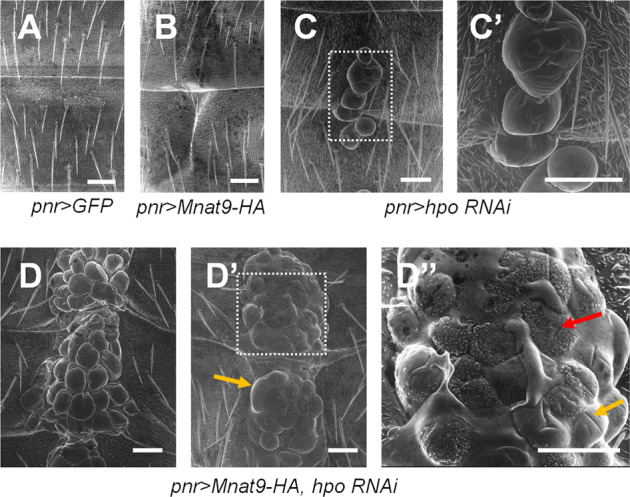
SEM images of tumorous tissue growth in the abdomen caused by Mnat9 overexpression and Hpo depletion. **A** Control. **B** Overexpression of *Mnat9* by *pnr-Gal4* causes mild cleft in the abdominal midline. **C**, **C**′ Knockdown of *hpo* induces a small number of tumorous tissues with relatively smooth surfaces in the medial part of the abdomen. **D**–**D**″ *Mnat9* overexpression combined with *hpo* knockdown induces massive tumorous tissues protruding from the abdominal midline. Overgrown tissues show either smooth surfaces (yellow arrow) or roughened surfaces with granular protrusions (red arrow). Scale bars: 50 μm.

Next, we used SEM to examine the more detailed morphology of the abdominal defects caused by Mnat9 overexpression and *hpo RNAi*. Abdominal midline with Mnat9 overexpression showed clefts with no tumorous growth (Fig. 6B). *hpo RNAi* flies showed mild overgrowth with a few clusters of large cell mass (Fig. 6C, C’). In contrast, SEM images of the overgrown tissues caused by Mnat9 overexpression plus *hpo RNAi* showed large bundles of irregular cell mass in the abdominal midline (Fig. 6D–D″). Some of these tissues show smooth surfaces (yellow arrow in Fig. 6D’, D″), while others have rough surfaces with numerous granular protrusions (red arrow in Fig. 6D″), although the basis for this difference in the surface morphology is currently unknown.

To further analyze the overgrowth phenotype, we examined whether these protruded tissues are Mnat9-overexpressing cells targeted by *pnr-Gal4*. In *pnr* > *GFP* control adult flies, GFP was hardly detected in the midline of the abdomen. This suggests that the levels of *pnr* > *GFP* might be too low or the signal was blocked by thick abdominal cuticle layers to be clearly detected (Fig. S6A). However, high levels of GFP were detected in the protruded cell clusters along the abdominal midline when Mnat9 was overexpressed in the *hpo RNAi* background. Hence, overgrowth seen in the abdomen appears to be due to cell-autonomous effects in the *pnr*-expressing cells (Fig. S6B, B’).

## Discussion

We have shown that Mnat9 is necessary for organ growth by modulating the Hippo pathway. Three kinds of genetic interactions were observed between *Mnat9* and Hippo pathway genes. First, *Mnat9 RNAi* phenotypes in two different organs are enhanced by overexpression of Hippo pathway components such as Ex, Hpo, and Wts (Figs. 1F, G, 3G, H). Second, overgrowth resulting from knockdown of Ex, Hpo, or Mats is partially suppressed by *Mnat9 RNAi* (Fig. 2), which supports a role of Mnat9 in negative modulation of the Hippo pathway. Third, the notched wing phenotype by *Mnat9 RNAi* in the wing margin is suppressed by knockdown of Hippo pathway genes, including *mer, ex*, and *kibra* (Fig. 3). Taken together, these data suggest that Mnat9 might act to inhibit the upstream of the Hippo pathway, including Mer, Ex, and Kibra. Consequently, reduced Mnat9 negatively affects Yki, in agreement with the suppression of the *Mnat9 RNAi* phenotype by Yki overexpression (Fig. 3C).

Our data show that Mnat9 co-immunoprecipitates with endogenous Mer in S2 cells (Fig. 3I). Because Mer, Ex, and Kibra function in a protein complex for Hippo signaling [36, 37], Mnat9 may be required to antagonize this complex, thus affecting their downstream pathway to promote organ growth. In fact, *Mnat9 RNAi* led to an increase of Mer protein level in the wing disc (Fig. 4), suggesting that Mnat9 might be involved in the downregulation of Mer to inhibit Hippo signaling. Interestingly, Mnat9 knockdown did not noticeably affect the protein level of Kibra and Ex (Fig. S3). These results suggest that knockdown of Mnat9 may interfere with a different pool of Mer without affecting the Mer–Ex–Kibra complex. However, it is worth noting that *Mnat9 RNAi* wing phenotype is not only suppressed by the knockdown of Mer but also by reducing Ex and Kibra (Fig. 3). Hence, Mnat9 acts antagonistically to Ex and Kibra, although it does not affect their levels. It is possible that increased Mer level resulting from *Mnat9 RNAi* may impair the function of Ex and Kibra rather than their levels. Further studies are necessary to understand the effects of reduced Mnat9 and increased Mer on the function of Ex/Kibra at the molecular level. Since Mnat9 and Mer are associated with microtubules [21, 33], it is an intriguing question whether microtubules are involved in the Mnat9 interaction with Hippo signaling.

Another important issue is whether Mnat9 is not only required for the inhibition of Hippo signaling but also is sufficient to inactivate the Hippo pathway to induce overgrowth. Overexpression of Mnat9 by *en*-*Gal4* did not induce obvious overgrowth or *ex-lacZ* expression in the targeted tissues (Fig. S4). Hence, Mnat9 alone appears to be insufficient to induce overgrowth in the wing. However, Mnat9 may interact with other factors to induce overgrowth in a tissue-specific manner. In support of this possibility, Mnat9 overexpression together with *hpo RNAi* driven by *pnr-Gal4* results in striking tumorous growth of epidermal cells in the abdominal midline (Figs. 5, 6).

In addition to the combined effects of *hpo* *RNAi* and Mnat9 overexpression, such overgrowth in the abdomen may also depend on an interaction between Hippo and other pathways. It is noteworthy that Mnat9 plays a role in cell survival during organ development by inhibiting JNK signaling [21]. As JNK activity is required for spreading and adhesion of imaginal cells at the dorsal midline to complete the closure of the thorax [38], it is possible that the abdominal midline defects by Mnat9 overexpression might be due to abnormal fusion of midline cells, as in the thorax. Because JNK signaling is known to interact with the Hippo pathway positively or negatively [39], it remains to be studied whether tumorous growth by combined effects of Mnat9 overexpression and reduced Hippo signaling involves an altered JNK signaling.

It has been suggested that Mnat9 functions in developing *Drosophila* are evolutionarily conserved since loss-of-function phenotypes of Mnat9 can be effectively rescued by expressing hNAT9 [21]. Our data also indicate that Mnat9 and hNAT9 share similar features in inducing tumorous tissue growth through interaction with Hippo signaling. Currently, the biological function of hNAT9 in humans is unknown. Based on the effects of genetic interaction between hNAT9 and Hippo signaling in the fly, it would be interesting to see whether upregulation of hNAT9 might cause tumorous growth in human tissues when combined with a compromised Hippo signaling.

## Supplementary information

Reproducibility checklist

Supplemental material for the manuscript

## Data Availability

All data are available in the paper or the supplementary materials; raw data or resources are available upon request.
