# Supplementary material for: Modulation of Hippo signaling by Mnat9 N-acetyltransferase for normal growth and tumorigenesis in Drosophila
Source: Cell Death Dis. 2022 Feb 2;13(2):101. doi: 10.1038/s41419-022-04532-2 (PMC8810759; doi:10.1038/s41419-022-04532-2)
Supplement: Supplementary file 2 — Supplemental material for the manuscript [file 41419_2022_4532_MOESM2_ESM.docx]

**Supplementary Figures (S1~S6)**

**
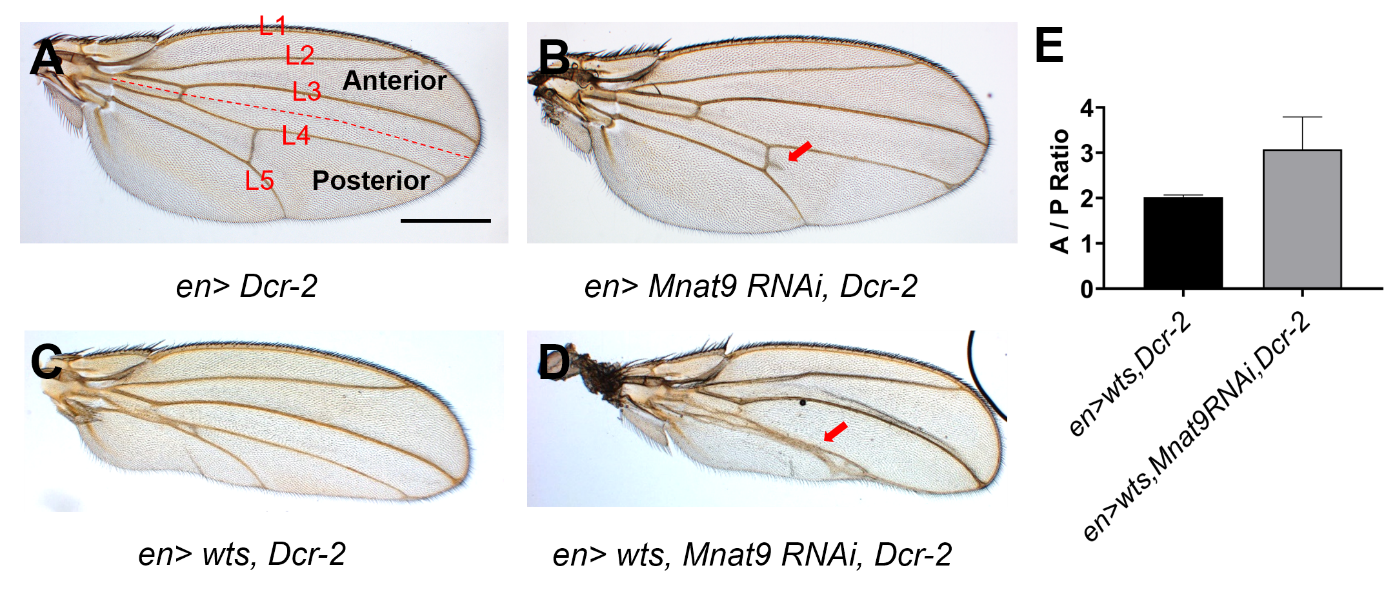
**

**Supplementary figure 1. Genetic interaction between *wts* and *Mnat9.*** (A) Control. Five longitudinal veins are labeled L1 through L5. The boundary between anterior and posterior compartments is shown as a red dashed line. (B) *Mnat9 RNAi* at 18^o^C shows a mild cross-vein phenotype (arrow). (C) Overexpression of *wts* causes a reduction in the posterior region of the wing. (D) *Mnat9 RNAi* enhances the phenotype of Wts overexpression shown in (C). The arrow in (D) indicates near-complete loss of the region between the veins L4 and L5. (E) The A/P boundary located between L3 and L4 veins was used to measure the A/P compartment size. The bar graph shows the quantification of data shown in (C) and (D). Scale bar: 0.5mm.

**
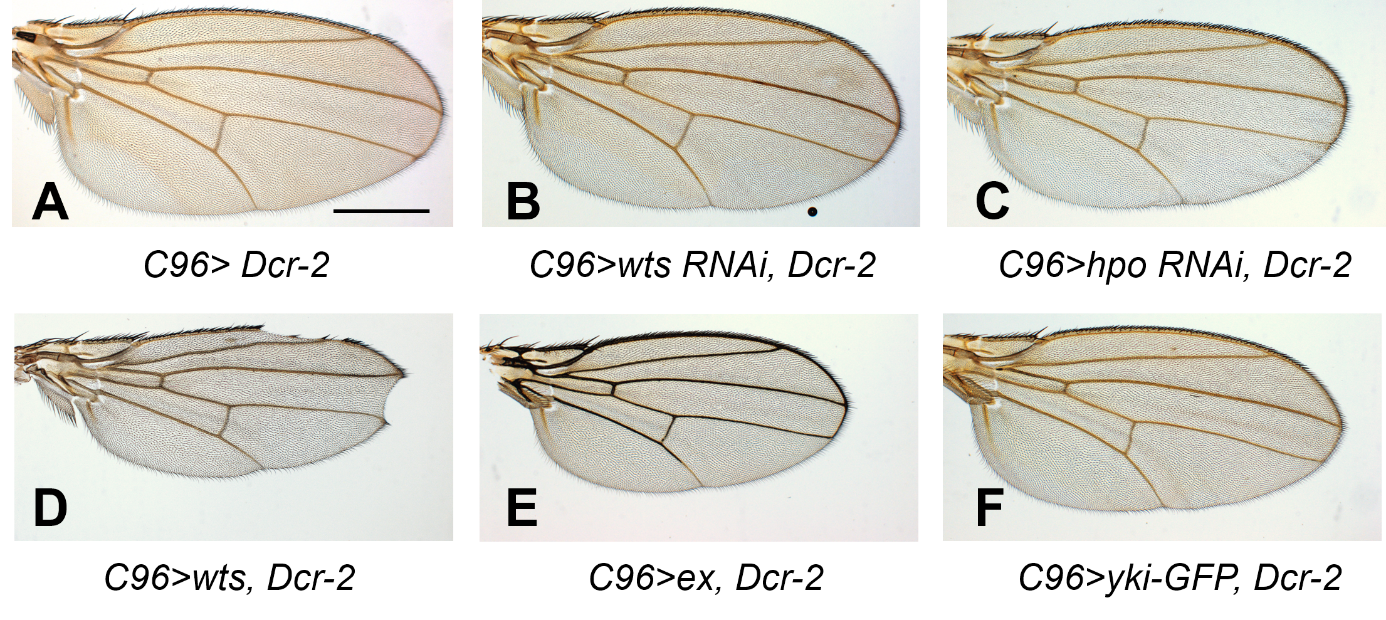
**

**Supplementary figure 2. Effects of overexpression or downregulation of Hippo pathway components using *C96-Gal4.*** (A) Control. (B) *wts RNAi* shows no notching phenotype*.* (C) *hpo RNAi* shows no notching*.* (D) Overexpression of Wts causes a small, notched wing. (E) Ex overexpression shows wing reduction but no notching. (F) Overexpression of Yki shows no notching. Scale bar: 0.5mm


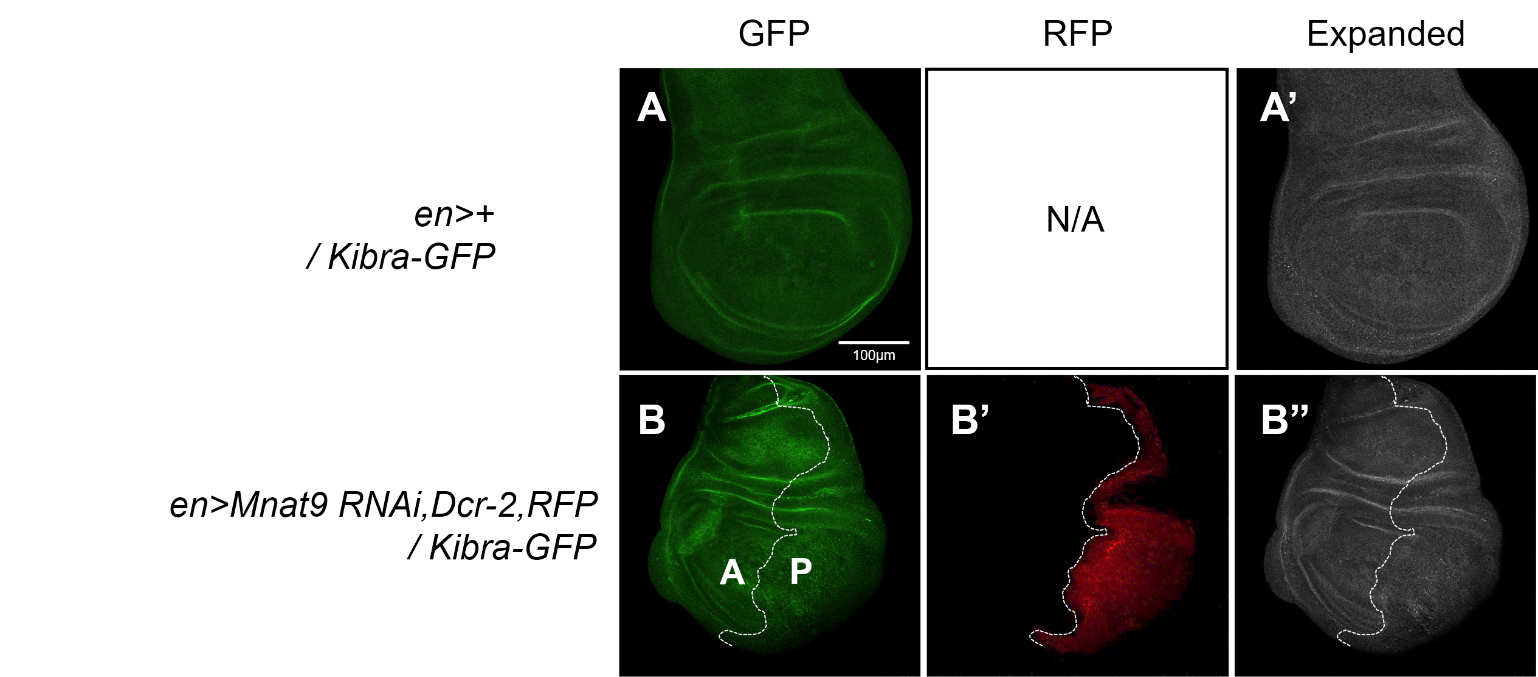


**Supplementary Figure 3. Mnat9 knockdown does not affect the level of Kibra and Ex.** (A, A’) Control wing disc. (B-B’’) Wing disc with *Mnat9 RNAi* and *Kibra-GFP*. Mnat9 knockdown in the RFP-positive posterior domain (B’) does not alter the level of Kibra-GFP (B) or Ex (B”). Scale bar: 100μm. Dashed line: Anterior (A)-Posterior (P) boundary.

**
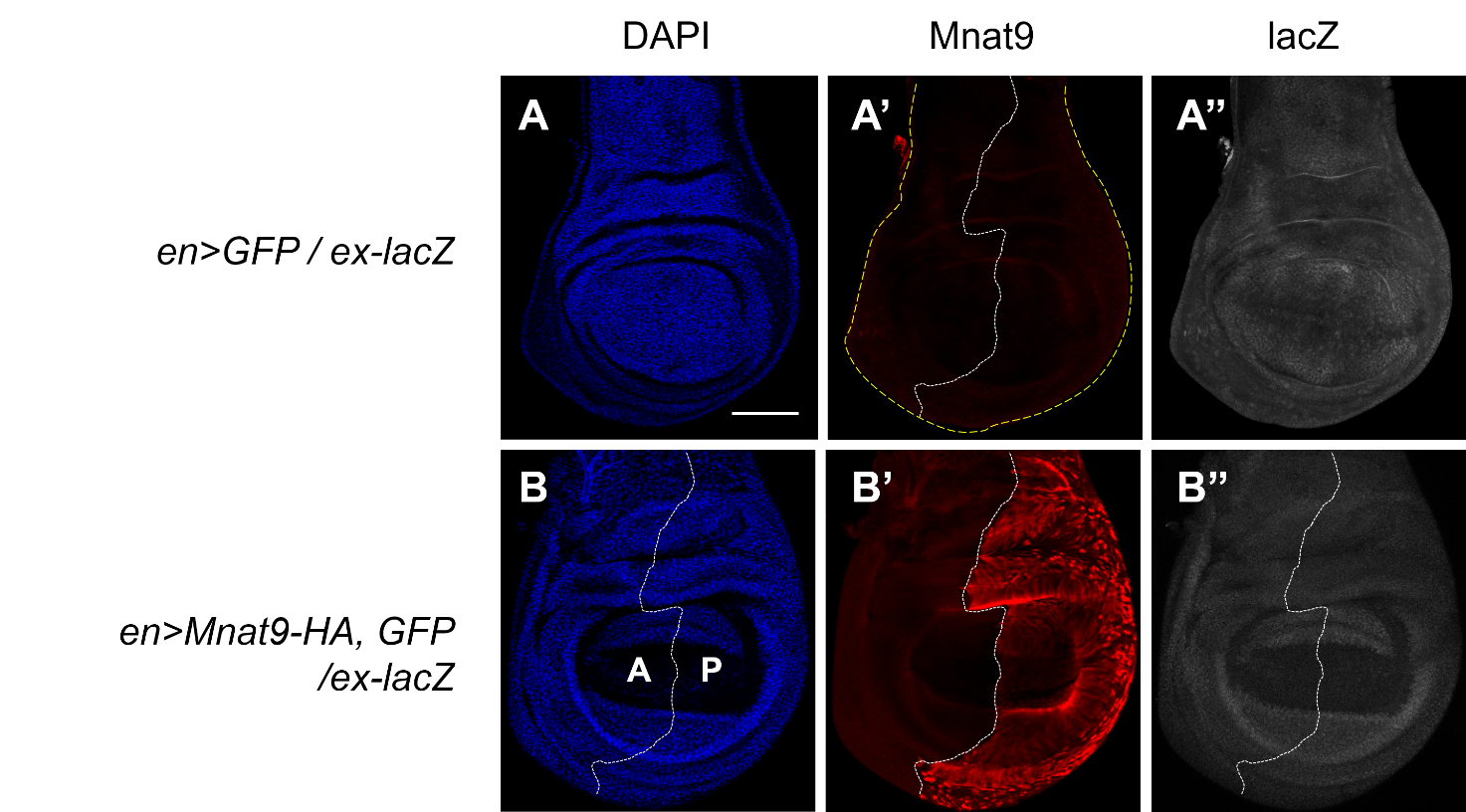
**

**Supplementary figure 4. Mnat9 overexpression does not change ex-lacZ level in the wing disc.** (A-A’’) *en>GFP/ex-lacZ*, Control. (B-B’’) *en>Mnat-HA, GFP/ex-lacZ*. Mnat9 overexpression (B’) by *en-Gal4* does not affect the *ex-lacZ* level (B”) in the posterior region of the wing disc. Scale bar: 100μm. Dashed line: Anterior-Posterior boundary.

**
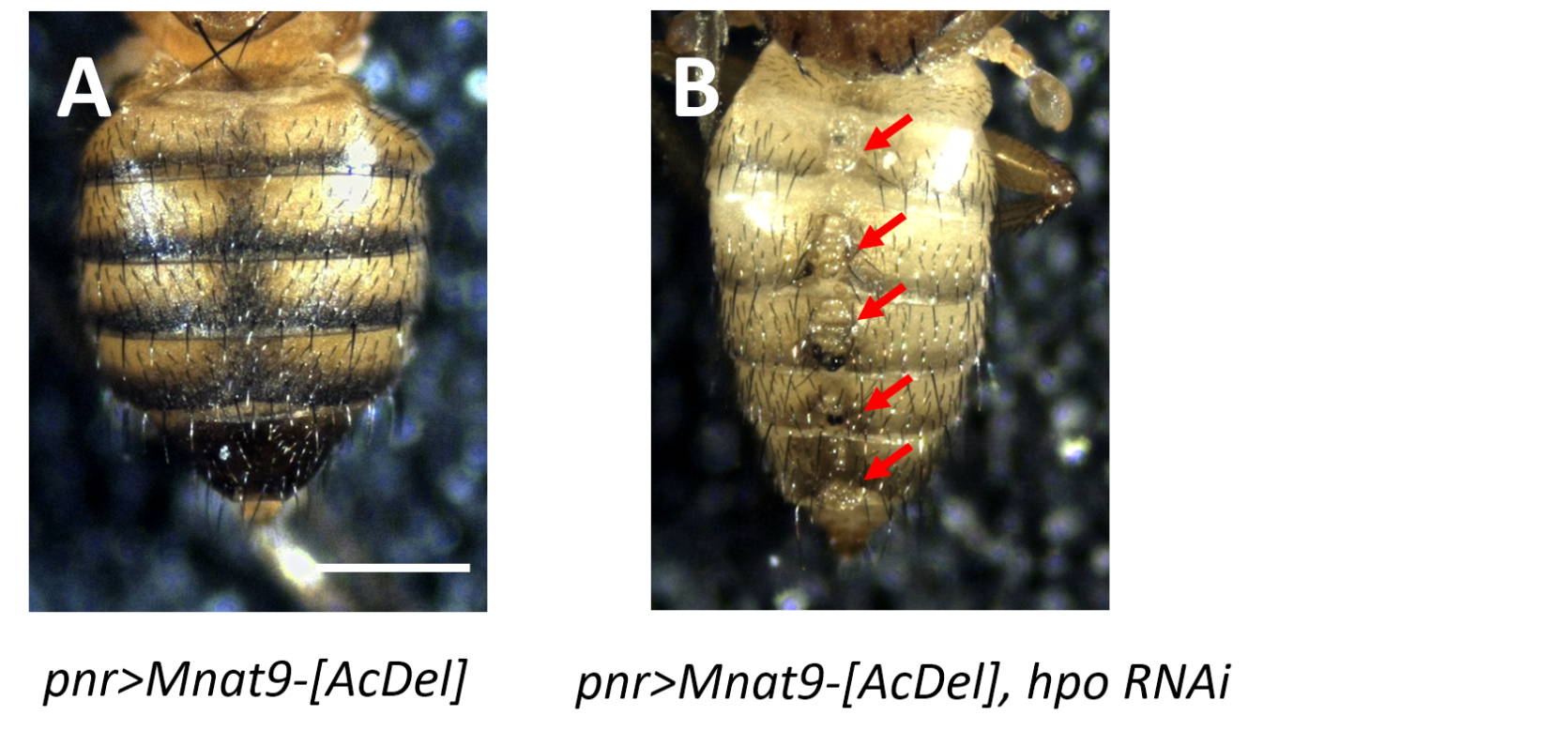
**

**Supplementary figure 5. N-terminal acetyltransferase activity is dispensable for genetic interaction between *Mnat9* and *hpo*.** (A) Overexpression of Mnat9-[AcDel] (a mutant form deleted in the acetyl CoA binding motif) shows no visible abdomen phenotype. (B) Overexpression of Mnat9-[AcDel] with *hpo RNAi* causes strong abdominal defects with overgrowth (arrows). Scale bar: 0.5mm.**
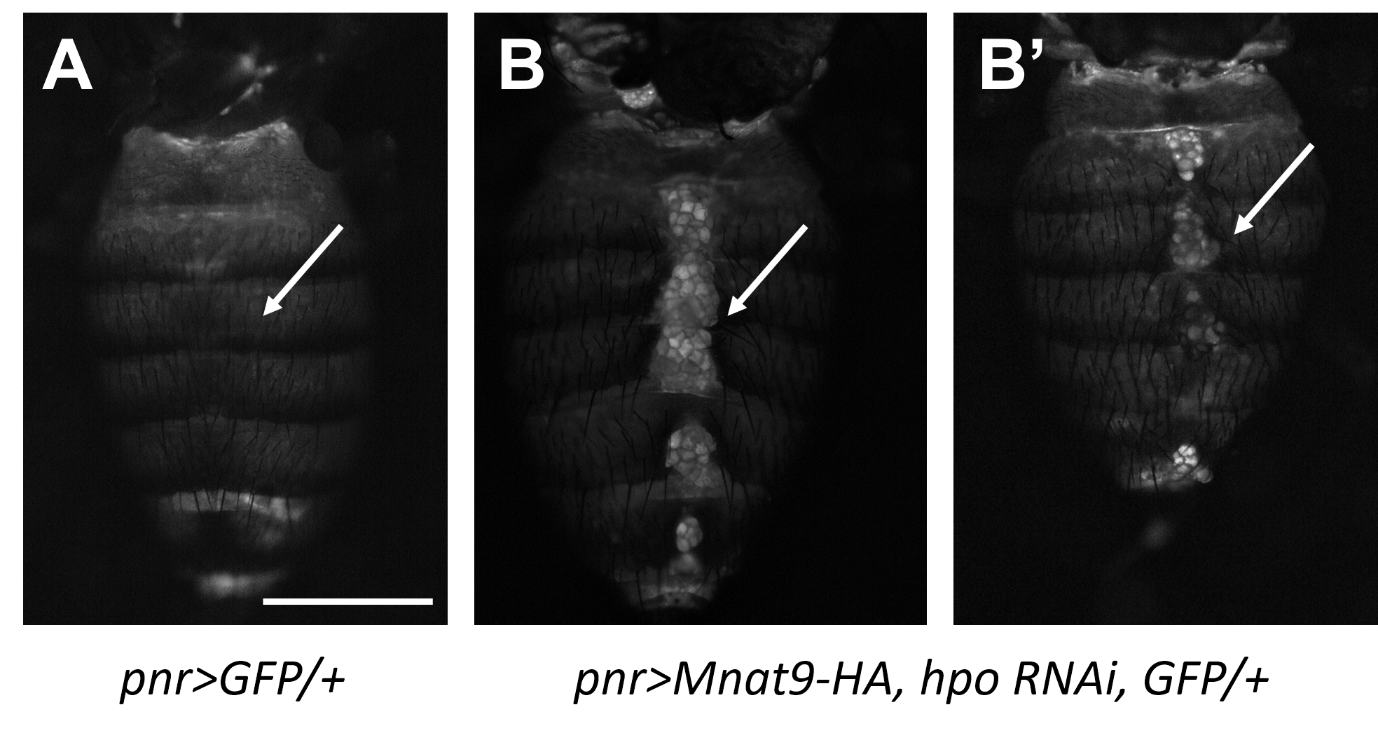
**

**Supplementary figure 6. Overgrown tissues caused by Mnat9-Hpo interaction show GFP expression*.*** (A) Control. GFP-positive cells are barely visible in the abdominal midline (arrow). (B, B’) Two examples of overgrown tissues showing GFP expression (arrows). Scale bar: 0.5mm.
